# Anesthetic Management for Inclusion Body Myositis in Coronary Artery Bypass Graft Surgery

**DOI:** 10.1155/2020/6679156

**Published:** 2020-12-24

**Authors:** Uoo Kim

**Affiliations:** Loma Linda University School of Medicine, Department of Anesthesiology, Division of Cardiothoracic Anesthesiology, Loma Linda, CA, USA

## Abstract

Anesthetic management for patients with certain neuromuscular disorders may be challenging due to contraindications to triggering agents secondary to increased susceptibility for malignant hyperthermia (MH). Inclusion body myositis (IBM) is an inflammatory muscle disease that causes concern for the anesthesiologist due to potential respiratory muscle weakness and hyperkalemia with succinylcholine. Elevated serum creatinine kinase levels found in IBM also raise the possibility of increased susceptibility to MH. This case report describes a successful anesthetic course with special considerations in a patient with IBM undergoing general anesthesia for coronary artery bypass grafting (CABG) under cardiopulmonary bypass (CPB) using total intravenous anesthesia (TIVA).

## 1. Introduction

Total intravenous anesthesia (TIVA) is indicated in patients with certain neuromuscular disorders that have contraindications to triggering agents due to increased susceptibility for malignant hyperthermia (MH). Inclusion body myositis (IBM) is an inflammatory muscle disease that causes concern for the anesthesiologist due to potential respiratory muscle weakness and hyperkalemia with succinylcholine. Sensitivity to neuromuscular blockade is a potential concern, with most clinicians using a smaller dose or avoiding muscle relaxants altogether. Elevated serum creatinine kinase levels found in IBM also raise the possibility of increased susceptibility to MH. This case report describes a successful anesthetic course in a patient with IBM undergoing general anesthesia for coronary artery bypass grafting (CABG) under cardiopulmonary bypass (CPB) using total intravenous anesthesia (TIVA) and neuromuscular blockade.

## 2. Case Report

A 66-year-old 76 kg male with IBM confirmed by muscle biopsy presented with symptoms of exertional throat tightness. Workup revealed multivessel coronary artery disease, and the decision was made to proceed with CABG. The patient had mild upper extremity weakness due to IBM without significant dysphagia or respiratory muscle involvement. Pulmonary function test results 4 months prior did not reveal any significant respiratory issues, with total lung capacity and vital capacity at 89% and 85% of the predicted value, respectively. Of note, the patient described a family member having a “bad reaction to anesthesia” requiring prolonged hospital stay. Unfortunately, the family member was unavailable for further questioning. Based on the history of IBM, elevated creatinine kinase, and potential family history of MH, a nontriggering TIVA was planned. Volatile anesthetics were physically removed from the ventilator circuit. Charcoal filters were applied to the inspiratory and expiratory valves and flushed with 100% oxygen. Intubating conditions were achieved with induction agents including propofol, fentanyl, and a lower intubating dose of rocuronium at 0.4 mg/kg. General anesthesia was maintained with a propofol and remifentanil infusion. Bispectral index (BIS) monitoring guided by clinical signs was utilized to guide adequate anesthetic depth throughout the case. The patient had return of four strong twitches using a peripheral nerve stimulator within one hour of induction. Rocuronium dose of 0.15 mg/kg was repeated every 60 minutes throughout the surgery with consistent recovery of twitch response prior to repeat administration. The surgery was uneventful, and the patient was transferredto the intensive care unit (ICU) intubated and sedated per institutional protocol. Reversal with sugammadex at 2 mg/kg was given upon arrival to the ICU. The patient was extubated within 1 hour after meeting extubation criteria and had no complaints of intraoperative awareness or new muscle weakness. After a smooth postoperative course, the patient was discharged home on postoperative day 3.

## 3. Discussion

Sporadic inclusion body myositis (IBM) is a rare muscle disorder associated with elevated serum creatine kinase (CK) levels and an abnormal electromyogram. It is the most common idiopathic inflammatory myopathy after age 50, clinically presenting with chronic proximal leg and distal arm asymmetric muscle weakness [1]. The pathophysiology is unknown, with evidence pointing towards a degenerative condition [2]. Treatment options differ from the other myopathies in its class, with failure to respond to traditional immunosuppressive therapy. Physical therapy has been shown to improve muscle strength [3]. However, in the majority of patients with IBM, prognosis is poor, with most patients developing progressive muscle weakness requiring support with a cane or wheelchair.

In severe cases, IBM may lead to respiratory muscle involvement causing decreased respiratory effort, with increased risk of pulmonary complications and prolonged ventilator support [4]. A potential treatment option described in one case study known as isokinetic inspiratory muscle training (IMT) may be beneficial. Initiation of IMT was associated with an increase in the strength of the inspiratory muscles resulting in successful weaning from mechanical ventilation [5]. Preoperative management should include assessment of a baseline pulmonary status with pulmonary function testing and proper consulting of neurology and rheumatology for optimization of treatment modalities.

Although there is lack of evidence for a direct correlation between IBM and MH, TIVA during the intraoperative course should be considered. Patients with IBM have elevated CK levels, which may indicate an increased susceptibility to MH. A Mayo Clinic study found that 49% of patients with elevated CK were found to be MH susceptible by the caffeine-halothane contracture test on muscle biopsies [6]. Britt et al. found 80% of MH survivors had increased CK levels [7]. Family history in addition to elevated CK increases the risk to 94% probability of having MH [8]. The main concern anesthesiologists have with TIVA is the potential for intraoperative awareness, especially during cardiac surgery. BIS monitoring guided by clinical signs is recommended to maintain an adequate depth of anesthesia during the case [9].

Neuromuscular blockade in patients with IBM does not appear to cause residual muscle weakness based on existing literature review. The largest case series examined 16 patients with IBM undergoing surgery at Mayo Clinic using both depolarizing and non-depolarizing agents with uneventful perioperative outcomes [10]. A separate case report describes a patient undergoing general anesthesia for video-assisted thoracoscopic surgery [11]. A small 10 mg dose of rocuronium was administered for induction with TIVA maintenance using propofol and opioids. Neostigmine was administered for reversal of neuromuscular blockade with return of four strong twitches on peripheral nerve monitoring. However, the patient required prolonged ventilator support in the ICU. This case highlights the need for thorough preoperative workup to assess for baseline muscle weakness from IBM which likely contributed to the prolonged postoperative ventilatory support, based on absence of residual neuromuscular blockade. Administering a decreased dose or avoiding paralytics altogether for shorter cases may be prudent due to decreased pulmonary function and potential for prolonged ventilatory support in certain patients with IBM, as the paralytics may cloud the picture of existing respiratory muscle weakness. For reversal of neuromuscular blockade, sugammadex is recommended as it has been shown to be associated with a lower incidence of major pulmonary complications [12].

Succinylcholine is relatively contraindicated in patients with other primary muscle diseases such as Duchenne or Becker muscular dystrophy because of an exaggerated hyperkalemic response and has not been studied closely in patients with IBM [13]. In the Mayo Clinic case series, succinylcholine was used for six patients with no evidence of dysrhythmia or electrocardiographic signs of hyperkalemia [10]. However, serum electrolyte levels were not measured. The limited number of patients and lack of objective data are inconclusive. A larger study is required for a definitive recommendation regarding depolarizing agents and their response in IBM.

## 4. Summary

IBM clinically presents with varying degrees of muscle involvement. It is important for the anesthesiologist to be aware that IBM may also affect the respiratory muscles in severe cases, which may lead to postoperative respiratory complications including prolonged mechanical ventilation. Recommendations include preoperative optimization, with judicious use of neuromuscular blockade, consideration for total intravenous anesthesia, and planning for potential prolonged ventilator support postoperatively depending on the severity of preexisting conditions due to IBM. Further studies are required for conclusive evidence regarding specific anesthetic agents, but with adequate planning and preparation, general anesthesia can be safely administered in those with IBM.
